# Epidemiological changes in tuberculosis and genotyping characteristics of *Mycobacterium Tuberculosis* in Ningxia, China

**DOI:** 10.3389/fmicb.2025.1582163

**Published:** 2025-06-04

**Authors:** Guangtian Liu, Jufen Lv, Linlin Chen, Yinglong Ma, Bofei Liu, Xuefeng Jiang

**Affiliations:** ^1^The Fourth People’s Hospital of Ningxia Hui Autonomous Region, Yinchuan, China; ^2^The College of Public Health, Ningxia Medical University, Yinchuan, China

**Keywords:** tuberculosis (TB), Beijing genotype, spatial clustering, epidemiological trend, prevention and control

## Abstract

**Introduction:**

China is one of the three countries with the largest TB burden globally, with an increased number of patients reported in 2021.

**Objective:**

In this study, we aimed to investigate the epidemiological profile of tuberculosis (TB) and the genotype characteristics of Mycobacterium tuberculosis (MTB) in the Ningxia Hui Autonomous Region, China.

**Methods:**

From 2005 to 2023, to provide a scientific basis for the precise prevention and control of TB. Epidemiological data on TB in Ningxia were obtained from the China Disease Control and Prevention Information System from 2005 to 2023. The temporal trend of TB incidence was assessed using a Joinpoint regression analysis (Joinpoint 5.2.0), and spatial autocorrelation analyses were performed using ArcGIS 10.8. Spoligotyping and McSpoligotyping based on 222 isolated MTB strains.

**Results:**

From 2005 to 2023, 51,345 patients with TB were reported in Ningxia. The incidence of TB decreased from 48.22/100,000 in 2005 to 30.47/100,000 in 2023. Joinpoint analysis showed that the incidence of TB in all age groups exhibited an overall decreasing trend. The incidences were significantly lower among urban residents than among rural residents. A spatial analysis showed that the southern mountainous area had a high incidence, with an average annual incidence of more than 60/100,000 in the Xiji, Lund, and Haiyuan counties, and this showed significant spatial clustering in 2007, 2009, 2014, 2016, and 2018. Genotyping showed that Beijing was the main genotype in Ningxia, accounting for 80.63% of the total (78.26% in 2005–2012 and 83.18% in 2013–2023). A cluster analysis showed that the Beijing type had strong intraregional transmission characteristics. The overall incidence of TB in Ningxia, China, showed a significant downward trend, but the prevalence was high in the southern mountainous regions and rural populations. The high aggregation of Beijing-type genotypes suggests a risk of intra-regional transmission and the need to strengthen surveillance and transmission chain analyses.

**Conclusion:**

TB incidence in Ningxia declined from 48.22 to 30.47/100,000 (2005–2023), yet remains high in southern mountainous regions. Persistent Beijing-type M. tuberculosis strains dominate, suggesting sustained transmission. Targeted interventions and further molecular studies are needed to enhance control in endemic areas.

## 1 Introduction

Tuberculosis (TB), caused by *Mycobacterium tuberculosis* (MTB), remains one of the leading causes of death from infectious diseases worldwide. In 2021 alone, an estimated 10.6 million new cases and 1.6 million deaths were reported globally (Organization, W.H, 2021). Although substantial progress has been made in reducing the TB incidence globally, significant regional disparities persist (Guo et al., 2023; Orgeur et al., 2024). China accounts for approximately 8.4% of global TB cases, with considerable geographic heterogeneity in the disease burden owing to socio-economic, environmental, and demographic factors (AlKhateeb et al., 2020; Chakaya et al., 2021). The western provinces, particularly the Ningxia Hui Autonomous Region, face disproportionately higher TB incidences, primarily owing to economic underdevelopment, the limited healthcare infrastructure, and high internal migration. With an annual incidence of 30–40 per 100,000—almost double the national average—Ningxia remains a TB hotspot. However, comprehensive molecular epidemiologic data on circulating MTB strains in the region remain limited, hindering targeted public health interventions.

Understanding MTB genotypic diversity is crucial for elucidating its transmission dynamics and designing region-specific control strategies (Qian et al., 2002; Wada et al., 2009). The Beijing genotype, predominant in East Asia, has been associated with increased virulence and drug resistance, and it plays a key role in sustaining transmission in high-burden areas (Castillos de Ibrahim das Neves et al., 2023; Dlamini et al., 2023). Whereas whole-genome sequencing (WGS) offers high-resolution insights into strain diversity and transmission chains, its widespread implementation in resource-limited settings is constrained by cost-associated and technical demands. Spoligotyping and its improved variant, McSpoligotyping, offer cost-effective and scalable alternatives, capable of lineage identification and international comparisons based on established databases (Napier et al., 2023).

The aim of this study was to conduct a comprehensive assessment of the temporal, spatial, and molecular epidemiology of TB in Ningxia from 2005 to 2023. By analysing the incidence trends, spatial distribution, and genotypic profiles of MTB strains (Yang et al., 2023), we seek to improve the understanding of local transmission patterns. Our findings will support the development of tailored control strategies in Ningxia and contribute to the broader global effort to map MTB diversity and enhance molecular surveillance in under-resourced regions.

## 2 Methods

### 2.1 Genotyping experiments

#### 2.1.1 Strain source and isolation

From 2005 to 2023, 222 MTB strains were isolated from patients treated at the Fourth People’s Hospital of the Ningxia Hui Autonomous Region. Owing to resource constraints for long-term strain preservation, the sample size was limited but representative of the major endemic areas of Ningxia, covering both southern counties with high incidences (e.g., Xiji) and northern districts with low incidences (e.g., Xingqing). Culturing and isolation were performed in the reference laboratory of the Fourth People’s Hospital of the Ningxia Hui Autonomous Region. The H37Rv standard reference strain was provided by the National Tuberculosis Reference Laboratory of the Chinese Centres for Disease Control and Prevention. In total, 222 isolates were successfully genotyped using spoligotyping and McSpoligotyping. This study was approved by the Ethics Committee of the Fourth People’s Hospital of Ningxia Hui Autonomous Region, with ethics review number 2025-QSY-005.

#### 2.1.2 Spoligotyping of strains

Spoligotyping approaches have been described previously (Zeng et al., 2016; Abebe et al., 2019; Yin et al., 2023; Getahun et al., 2024). Briefly, we used the standard 43 spacer oligonucleotides for the dot-detection standard protocol. The PCR amplification conditions were as follows: 96°C for 3 min, 96°C for 1 min, 55°C for 1 min, 72°C for 30 s, for a total of 35 cycles, followed by 72°C for 5 min. For membrane preparation, each oligonucleotide corresponded to a spacer-region sequence between the direct repeat (DR) sites. For hybridisation and detection, 20 μL of the polymerase chain reaction (PCR) product was added to 150 μL of 2*SSPE/0.1% sodium dodecyl sulphate, heated and denatured at 100°C for 10 min, diluted, and added to the notch; this was hybridised with the membrane at 55°C for 60 min, and the membrane was washed and incubated at 42°C or 30°C with a horseradish peroxidase streptavidin–conjugate. Membrane detection was performed using a CDP star and exposed to an X-ray film for 5 min.

#### 2.1.3 McSpoligotyping of strains

McSpoligotyping was performed as described previously (Zeng et al., 2018; Pan et al., 2021). Briefly, PCR amplification and melting curve analysis were performed as one program and completed continuously on a SLAN48P real-time fluorescence PCR instrument. This system is a three-tube four-colour system, which means that for each sample, three tubes are needed for detection, and in total, 43 spacers are detected. The 25 μL reaction system included the following: McSpoligotyping PCR Mix (A/B/C), 19.75 μL; McSpoligotyping enzyme mixture, 0.25 μL; and the sample to be tested/negative control/positive control, 5 μL. The reaction conditions were as follows: 50°C for 5 min → 95°C for 10 min → (95°C for 15 s → 57°C for 15 s → 72°C for 5 s) × 50 cycles. The melting curve program was set as follows: 95°C for 1 min → 35°C for 1 min → 3°C–90°C with a heating rate of 0.04°C/s for melting analysis, and during this stage, fluorescence signals from the FAM, ROX, and CY5 channels were collected.

#### 2.1.4 Clustering analysis of genotypes

The spoligotyping results were converted into 43-bit binary strings (‘1’ indicates the presence of spacer sequences, and ‘0’ indicates their absence), and invalid data owing to PCR amplification failure or ambiguous signals were excluded to ensure data integrity. The pre-processed binary data were compared with the SITVIT2^1^ database^2^ (Brudey et al., 2006) to obtain Spoligotype International Type (SIT) numbers and lineage information to form a complete strain characterisation dataset. Using the Spoligotyping module of the MIRU-VNTRplus online platform^3^ (Weniger et al., 2012), we imported the binary data files, calculated the pairwise genetic distances between strains based on the Jaccard distance to generate the symmetric distance matrix, and constructed the minimum generative distance matrix based on the Kruskal algorithm. Using the Kruskal algorithm to construct the minimum spanning tree (MST) and according to the classical definition of the MTB clonal complex (CC; Weniger et al., 2010), the maximum spacer sequence divergence threshold was set to 1, and the strains with divergence ≤1 were classified as the same CC. Hierarchical Spring Embedder was used to optimise the MST layout to reduce cross-links, and node positions were manually adjusted when necessary. To verify the topological stability, 1,000 bootstrap analyses were performed, and branches with ≥70% support were considered plausible.

### 2.2 Data sources, cleaning, processing, analysis, and visualisation

Epidemiological data on TB were obtained from the China Information System for Disease Control and Prevention. Data cleaning and processing and statistical analyses were performed using Excel 2020 with a focus on variables such as sex, age, diagnostic indicators, and treatment information. Age groups were classified into three categories (0–14, 15–64, and ≥65 years) based on the statistical yearbook published by the Ningxia Statistics Bureau.

Subsequently, Joinpoint regression analysis (Version 5.2.0, Information Management Services Inc., Calverton, MD, United States) was used to fit the data into a series of segmented linear regression models connected by “joinpoints” (Li Huizhang, 2020). This approach was used to analyse temporal trends in the time-series data and characterise the epidemiological features of TB in the region. Additionally, spatial autocorrelation analysis was performed using ArcGIS 10.8 (Esri, Redlands, CA, United States) to preliminarily explore the spatial epidemiological patterns and geographical distribution of TB in the region.

## 3 Results

### 3.1 Demographic trends

During 2005–2023, 51,345 patients with TB were reported in the Ningxia Hui Autonomous Region. The patients had an average age of 50.07 ± 20.53 years (0–10), including 29,330 males and 22,015 females, with a male-to-female ratio of 1.331:1. Further, 37,410 (72.86%) were Han, 13,811 (26.90%) were Hui, and 124 (0.24%) were from other ethnic groups; 19,835 (38.63%) were urban residents and 31,388 (61.13%) were rural residents, with 122 (0.24%) having unknown addresses. The distribution of occupations was mainly farmers, students, retirees, and those engaged in housework or unemployed, with farmers accounting for 66.05% (Table 1). Notably, the average delay in seeking medical care was 62.6 ± 179.96 days; 42.47% were referred, 37.54% sought care directly, and the rest came from tracing, recommendations, health check-ups, or screenings. Most patients (94.98%) were newly diagnosed, whereas 5.02% were retreatment cases; 53.19% tested pathogen-positive, 43.52% tested negative, and a small proportion had extrapulmonary TB or missing results. Among pathogen-positive patients, the cure rate was 79.27%, whereas some experienced adverse reactions, treatment failure, or death; 83.86% received the standard 2HRZE/4HR regimen, with the others following alternative treatments (Table 1).

**Table 1:** Characteristics of TB patients in Ningxia from 2005 to 2023.

Characteristic	Total (*n* = 51,345)
Age, years	
Mean	50.07
SD	20.53
Sex, n (%)	
Women	22,015 (42.88)
Men	29,330 (57.12)
Nation, n (%)	
Han ethnic group	37,410 (72.86)
Hui Islamic ethnic group living across China	13,811 (26.90)
Other ethnic groups	124 (0.24)
Urban and rural distribution, n (%)	
Urban residents	19,835 (38.63)
Rural residents	31,388 (61.13)
Unknown address	122 (0.24)
Occupational groups, n (%)	
Farmers	33,914 (66.05)
Domestic and non-working	4,872 (9.49)
Students	3,456 (6.73)
Other occupations	9,103 (17.73)

**Figure 1:**
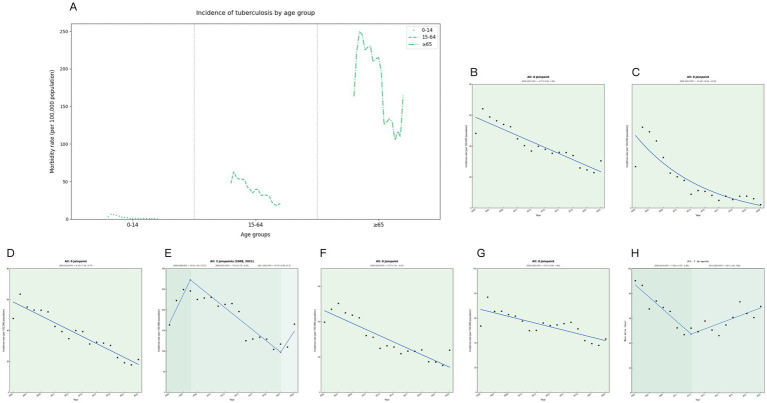
Trends in tuberculosis incidence in Ningxia from 2005 to 2023. **(A)** Tuberculosis incidence rates by age group for each year. **(B)** Trend chart of changes in tuberculosis in the whole population. **(C)** Trend chart of tuberculosis changes in 0–14 years old. **(D)** Trend chart of changes in tuberculosis from 14 to 64 years of age. **(E)** Trend map of tuberculosis changes at 65 years and above. **(F)** Trend chart of tuberculosis among urban residents. **(G)** Trends in tuberculosis among rural residents. **(H)** Trends in tuberculosis treatment delays.

The joinpoint regression analysis showed that the overall trend of the registered incidence of TB in Ningxia decreased from 2005 to 2023, and the registered incidence of TB in Ningxia showed a significant downward trend, with an average annual decrease of 4.77% (APC = −4.77, 95% CI: −5.99 to −3.56%, *p* < 0.05), decreasing from 48.22 to 30.47 per 100,000 (Figure 1B). From 2005 to 2023, the prevalence varied by age group, with the highest prevalence in the 65 and older age group, followed by the 15–64 age group, and the lowest prevalence in the 0–14 age group (Figure 1A). TB incidence in Ningxia showed a significant decline across all age groups. The most notable decrease was among children aged 0–14, with an annual reduction of 13.46% (*p* < 0.001) (Figure 1C). In the 15–64 age group, the incidence decreased by 6.12% annually (*p* < 0.001) (Figure 1D), whereas in those 65 and older, the decline was minimal and not statistically significant (*p* = 0.342) (Figure 1E). Urban and rural trends also differed, with the urban TB incidence decreasing by 5.77% annually (Figure 1F), a significantly greater reduction than the 2.61% annual decline in rural areas (*p* < 0.05) (Figure 1G). During this period, the average treatment delay for TB in the region decreased by 1.43% annually (*p* = 0.004). The most significant improvement occurred between 2005 and 2013, with an annual reduction of 7.38% (*p* < 0.05). However, from 2013 to 2023, treatment delays increased by 3.61% per year (*p* < 0.05), indicating a concerning reversal in progress (Figure 1H).

**Figure 2:**
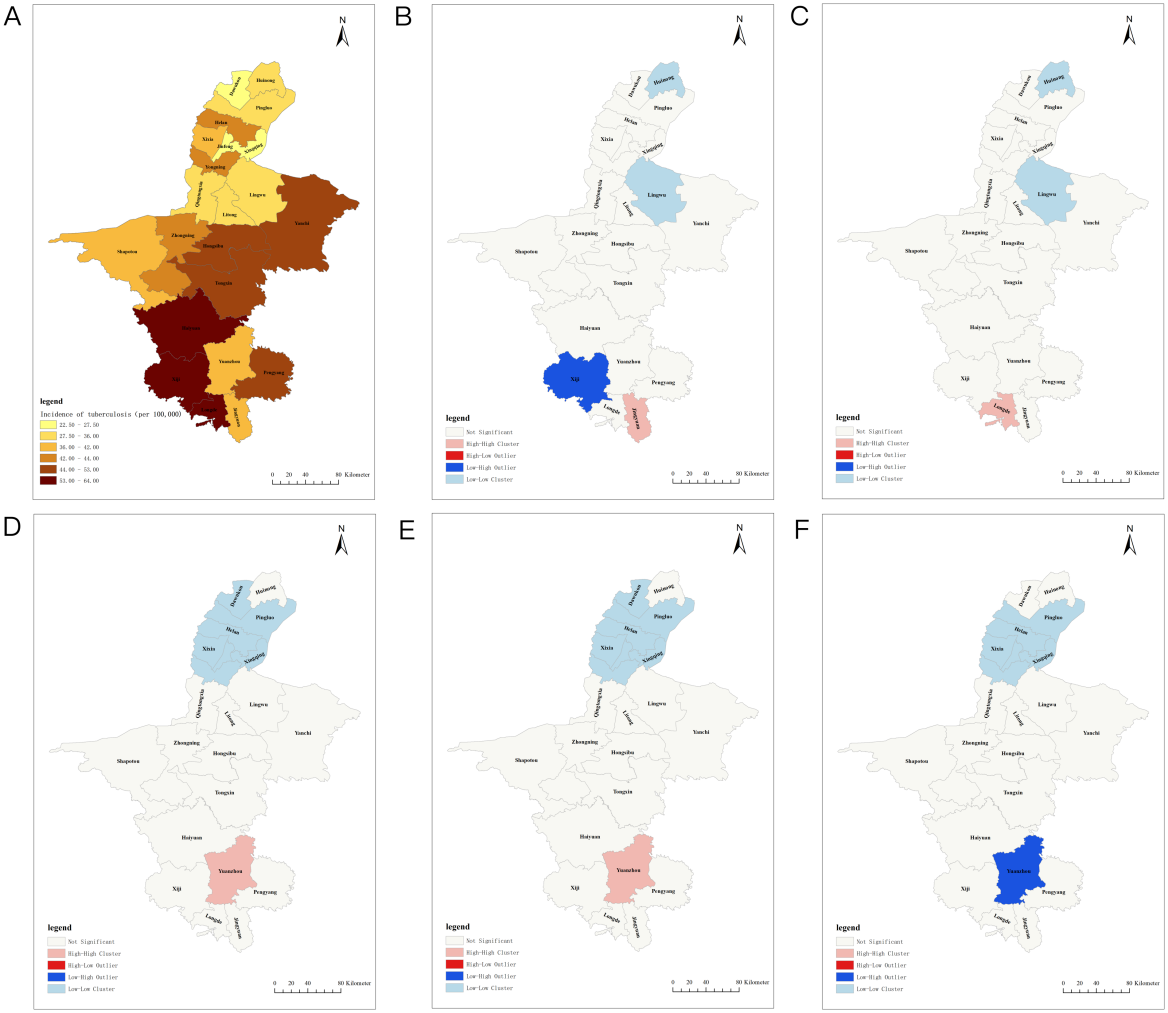
Spatiotemporal distribution of tuberculosis incidence in Ningxia. **(A)** Distribution of tuberculosis incidence in Ningxia. **(B)** Hotspot analysis of tuberculosis incidence in Ningxia in 2007. **(C)** Hotspots of tuberculosis incidence in Ningxia in 2009. **(D)** Hotspots of tuberculosis incidence in Ningxia in 2014. **(E)** Hotspot analysis of tuberculosis incidence in Ningxia in 2016. **(F)** Hotspots of tuberculosis incidence in Ningxia in 2018.

### 3.2 Spatial analyses

From 2005 to 2023, the TB incidence varied significantly across regions. Xingqing District had the lowest average annual incidence at 2.50/100,000, whereas Xiji County had the highest at 64.07/100,000. Three northern plain areas—Xingqing, Jinfeng, and Dawukou Districts—maintained rates below 30/100,000, whereas three southern mountainous areas—Xiji, Longde, and Haiyuan Counties—recorded rates exceeding 60/100,000. The spatial analysis showed fluctuations in Moran’s I index (−0.057 to 0.458), with significant TB clustering in 2007, 2009, 2014, 2016, and 2018 (*p* < 0.05). High–high clustering was concentrated in southern regions, such as Yuanzhou, Longde, and Jingyuan, whereas low–low clustering was observed in northern regions, including Lingwu, Huinong, Panglao, and Helan (Figure 2; Table 2).

**Table 2:** Global spatial autocorrelation of TB incidence in the Ningxia, 2005–2023.

Year	Moran’s I	Expected index	Variance	Z-Score	*p-*value
2005	0.232	−0.048	0.023	1.836	0.066
2006	0.153	−0.048	0.023	1.314	0.189
2007	0.249	−0.048	0.022	1.991	0.046
2008	0.172	−0.048	0.023	1.439	0.150
2009	0.275	−0.048	0.024	2.093	0.036
2010	0.213	−0.048	0.024	1.688	0.091
2011	0.062	−0.048	0.024	0.712	0.476
2012	−0.041	−0.048	0.023	0.045	0.964
2013	0.132	−0.048	0.022	1.202	0.229
2014	0.401	−0.048	0.021	3.087	0.002
2015	0.239	−0.048	0.022	1.934	0.053
2016	0.458	−0.048	0.021	3.455	0.001
2017	0.069	−0.048	0.024	0.761	0.446
2018	0.226	−0.048	0.019	2.009	0.045
2019	0.119	−0.048	0.024	1.077	0.282
2020	0.181	−0.048	0.024	1.479	0.139
2021	−0.057	−0.048	0.023	−0.062	0.950
2022	0.072	−0.048	0.023	0.791	0.429
2023	0.038	−0.048	0.024	0.557	0.577

TB, Tuberculosis.

### 3.3 Molecular findings

Genotyping revealed distinct shifts in the MTB lineage distribution between 2005–2012 and 2013–2023. During 2005–2012, the traditional spoligotyping of 115 strains identified 22 genotypes, with the Beijing family predominating (78.26%, 90/115). Among these, 11 genotypes matched known SIT codes, whereas 12 strains formed 11 novel genotypes (10.43% of the total). Non-Beijing strains (21.74%) included T1 (7.83%, 9/115), MANU2 (2.61%, 3/115), and U (0.87%, 1/115) lineages. From 2013 to 2023, the McSpoligotyping of 107 strains resolved 18 genotypes, showing increased Beijing family dominance (83.18%, 89/107) and reduced novel genotype detection (4.67%, 5/107). Non-Beijing strains (16.82%) comprised T (11.1%, 12/107) and U (0.93%, 1/107) lineages, with no MANU2 isolates detected (Table 3).

**Table 3:** Genotype distribution of 222 MTB isolates in the Ningxia region.

2005–2012 (*n* = 115)				2013–2023 (*n* = 107)			
Genotyping	SIT	Number	Spoligotype	Genotyping	SIT	Number	McSpoligotype
Beijing	1	82	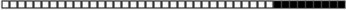	Beijing	1	83	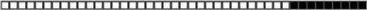
	1,364	1	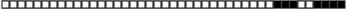		621	1	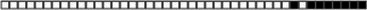
Beijing-Like	269	5	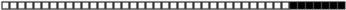		190	3	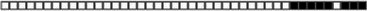
	796	1	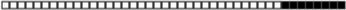		2,979	1	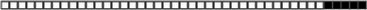
	406	1	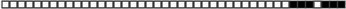		265	1	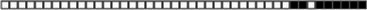
T1	53	2	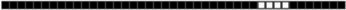	T1	53	5	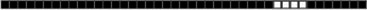
	334	6	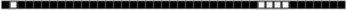		334	2	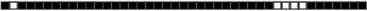
	393	1	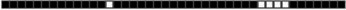		7	1	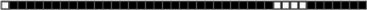
MANU2	54	2	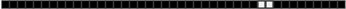		3,228	1	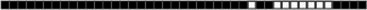
	1,192	1	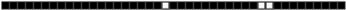		2,905	1	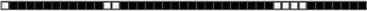
U	1,462	1	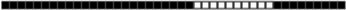	T2	848	1	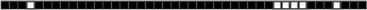
New	–	2	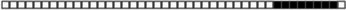	T3	37	1	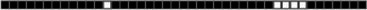
	–	1	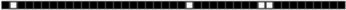	U	3,109	1	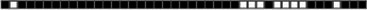
	–	1	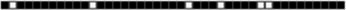	New	–	1	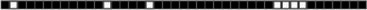
	–	1	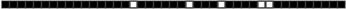		–	1	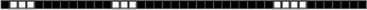
	–	1	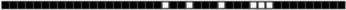		–	1	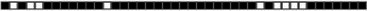
	–	1	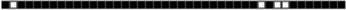		–	1	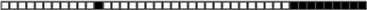
	–	1	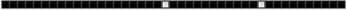		–	1	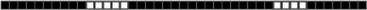
	–	1	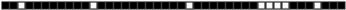				
	–	1	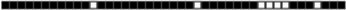				
	–	1	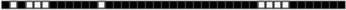				
	–	1	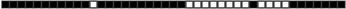				

**Figure 3:**
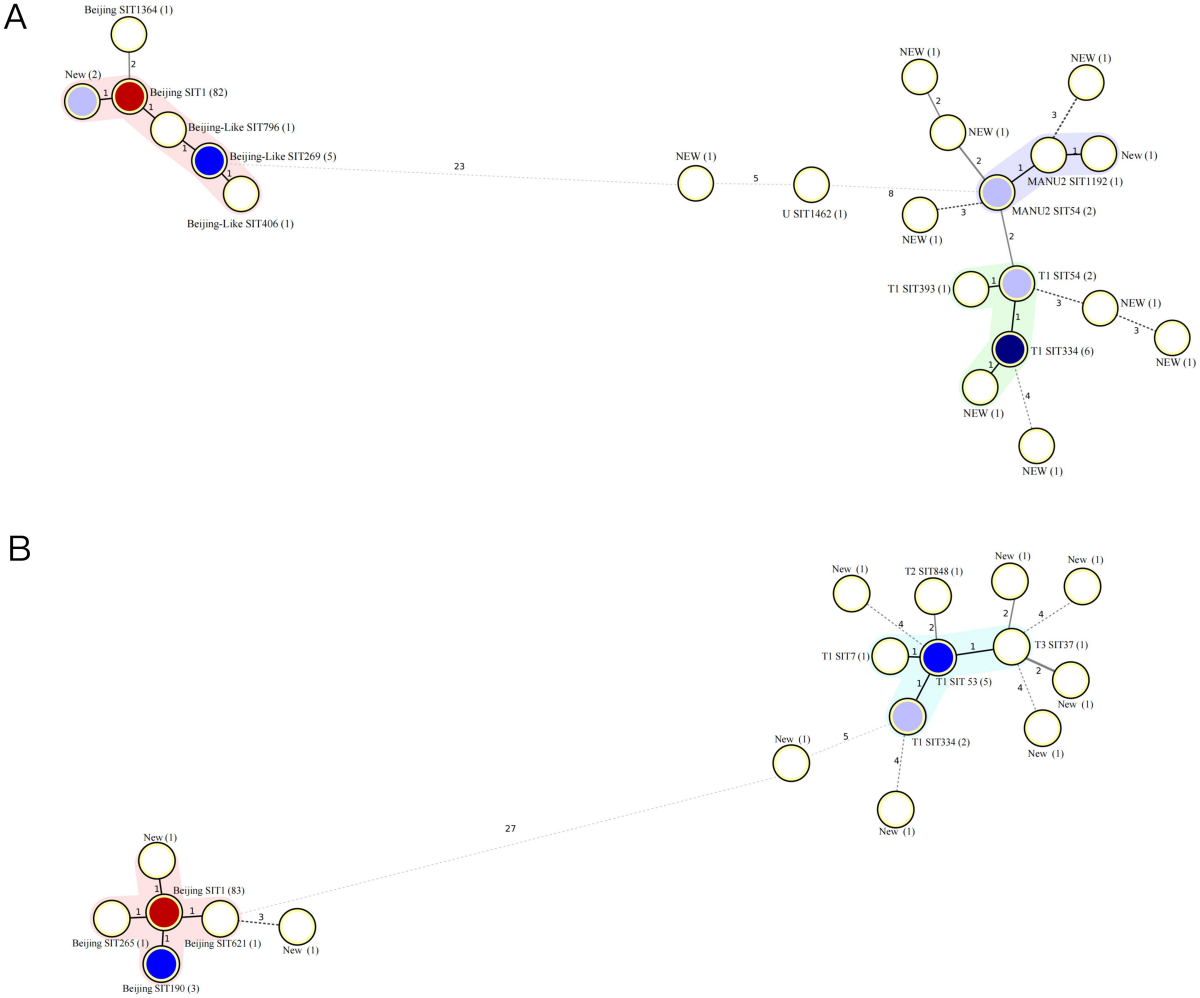
Minimum spanning tree of MTB isolates. **(A)** Minimum spanning tree of 115 MTB isolates from 2005 to 2013. **(B)** Minimum spanning tree of 107 MTB isolates from 2013 to 2023. MTB, *Mycobacterium tuberculosis.*

The cluster analysis of MTB strains using spoligotyping and McSpoligotyping revealed distinct transmission patterns. The spoligotyping of 115 isolates identified three major clusters, Beijing (79.13%, 91/115), MANU2 (3.48%, 4/115), and T (8.70%, 10/115), with 8.70% (10/115) as unique genotypes (clustering rate: 91.0%). Within the Beijing cluster, SIT1 dominated (90.11%, 82/91), followed by SIT269 (5.49%), and rare subtypes (NEW/SIT796/SIT406). The MANU2 cluster included SIT54 (*n* = 2), SIT1192 (*n* = 1), and an unclassified strain, whereas the T cluster comprised SIT334 (*n* = 6), SIT54 (*n* = 2), S393 (*n* = 1), and a novel genotype (Figure 3A). In contrast, the McSpoligotyping of 107 isolates resolved two lineages, Beijing (83.18%, 89/107) and T (8.41%, 9/107), with 8.41% (9/107) as unique genotypes (clustering rate: 91.59%). The Beijing lineage exhibited higher homogeneity, with the predominance of SIT1 increasing to 93.26% (83/89), alongside rare subtypes (SIT190/SIT265/SIT621/NEW). The T lineage diversified into SIT3 (*n* = 5), SIT334 (*n* = 2), and SIT7 (*n* = 1 each; Figure 3B).

## 4 Discussion

We observed that the patients were mainly middle-aged and older adults from 2005 to 2023 in Ningxia, China, similar to observations in most other provinces in the country. Farmers accounted for most patients, at 66.05%. China is one country with a high burden of TB, which has a slow onset and a long course, causing great harm to people. With the continuous introduction of new anti-TB drugs, the implementation of various prevention and control measures, and the substantial investment of funds, the epidemic has been controlled, exhibiting a downward trend (Chen et al., 2024; Tao Li et al., 2024). In 2005, the Ningxia Hui Autonomous Region started to use a unified online reporting system to manage TB. Since then, the incidence of TB has declined. The reported incidence of TB significantly decreased from 2005 to 2012, from a peak of 64.15 per 100,000 in 2006 to 40.36 per 100,000 in 2012. TB control in the Ningxia Hui Autonomous Region was effective during this period. On the one hand, during this period of rapid economic development, the level of medical technology significantly improved, curbing the spread of TB to a certain extent. On the other hand, the improvement in public health awareness and living conditions is crucial (Zhang et al., 2020). In the following years, from 2012 to 2019, the reported incidence remained between 30 and 40 per 100,000, which we refer to as a stable period. However, owing to the comprehensive outbreak of Coronavirus Disease 2019 (COVID-19) in China in 2020, changes in social management methods, lifestyles, and medical services led to a temporary decrease in the reported incidence of TB to <30 per 100,000 (Zhang et al., 2023).

Delays in seeking medical care remain a serious issue (Li et al., 2013). This situation slightly improved from 2005 to 2013, but since 2013, there has been a rebound in delays in seeking medical care, and methods to find patients have gradually changed from passive to proactive. With social developments, the epidemiological characteristics of TB are changing. From the perspective of its geographical distribution, the western side of the southern mountainous area had a high incidence of TB, with an annual average incidence of more than 60/10,000, indicating clustering characteristics. The southern mountainous area, represented by “Xi Hai Gu,” was identified by the United Nations Food Development, in 1972, as one of the 22 “least suitable areas for human habitation” in the world (Organization, W.H, 2015). Owing to the harsh living conditions, residents lack knowledge regarding TB and have poor nutrition, leading to a continuously high incidence of TB in this area. TB is closely linked to poverty. This situation is gradually improving; however, this area remains a key focus from the perspective of disease prevention and control (Wang et al., 2021).

The Beijing genotype is the predominant MTB genotype worldwide, particularly in China, where it has an advantage over other genotypes. Studies have reported that the Beijing genotype accounts for approximately 83.3% of the MTB strains in Beijing (Liu et al., 2018) and 73.2% nationwide (Wan et al., 2011). Our study showed that the proportion of the Beijing genotype among MTB strains in Ningxia is 80.63%. In Northern provinces, such as Shaanxi, the Beijing genotype is dominant (Li et al., 2020). In contrast, southern regions, such as Hainan and Guangxi, exhibit a lower prevalence of the Beijing genotype, with Guangxi showing significant diversity of MTB strains, including early branching strains of the Beijing lineage (Wan et al., 2011). This indicates a high prevalence of patients infected with the Beijing MTB genotype in Ningxia. The T and U families of the genotypes were present in Ningxia. The results of the MTB clustering analysis showed a high rate of clustering of the bacteria, indicating a certain degree of relatedness, and they may be mainly spread within the region; however, whether there is spatial overlap and an epidemiological link among patients needs further verification (Liu et al., 2018).

According to our results, we recommend improving awareness campaigns and educational initiatives to improve self-protection awareness among farmers and high-risk groups. Enhancing communication and cooperation within departments, such as the education department, should be the focus, including promoting a healthy lifestyle to reduce the incidence of TB (Chen et al., 2023). Furthermore, in terms of the treatment and management of patients with TB, strict control should be exercised over patients continuing to engage in work that may spread the disease, and patients should wear masks in crowded areas (Liu et al., 2024). Additionally, the follow-up of patients after short-term chemotherapy should be strengthened to prevent further spread. Moreover, targeted prevention and control should be implemented, including publicity activities in cooperation with local religious organisations, to ensure that local people participate in monitoring and control while respecting cultural differences.

The findings of this study align with observations from other regions with high TB burdens, providing a valuable comparative context. For example, studies conducted in Latin American countries, such as Mexico, revealed the high diversity of circulating MTB strains and complex transmission dynamics, underscoring the challenges faced in controlling the disease in similar socio-economic contexts (Mejía-Ponce et al., 2023). Research from Sub-Saharan Africa has similarly highlighted the prevalence and persistence of dominant strains, especially those resistant to multiple drugs, contributing to ongoing transmission despite targeted interventions (Ektefaie et al., 2021). Likewise, in Southeast Asia, specifically Vietnam, persistent high rates of drug resistance and the effectiveness of molecular surveillance strategies underline the critical importance of accurate genomic data to inform TB-control policies (Huong et al., 2024). In East Asia, a decade-long molecular epidemiological analysis in Japan’s Chiba Prefecture revealed the sustained dominance of the modern Beijing genotype, reflecting patterns similar to those observed in Ningxia and highlighting the need for region-specific genotypic surveillance (Kikuchi et al., 2022).

The spoligotyping and McSpoligotyping methods employed in this study remain important tools, because of their cost-effectiveness and convenience, and are particularly suitable for large-scale surveillance in resource-limited regions; for example, one study found that spoligotyping was the most widely used method in Latin America, accounting for 60.9% of the total (Castillos de Ibrahim das Neves et al., 2023). However, their limitations in accuracy and strain discrimination must be acknowledged. One study showed that spoligotyping can be used for low-resolution monitoring and WGS for higher-resolution studies (Napier et al., 2023). Some studies have documented discrepancies between spoligotyping and WGS, suggesting that spoligotyping may lack sufficient resolution to reliably distinguish between closely related strains, potentially leading to the misinterpretation of transmission dynamics (Ramazanzadeh et al., 2020; Bakuła et al., 2023). Thus, based on previous work, we continue to use spoligotyping and McSpoligotyping for sequencing. In the future, we will use WGS to compare the results of the two methods and for further verification. Although this study presents novel findings, there are certain limitations that need to be considered. First, the molecular typing of representative strains is required to better understand the epidemiological transmission patterns and evolutionary characteristics of the strains. Second, the number of strains isolated from patients was limited, indicating that future studies should include a larger sample size to validate the results of this study.

## 5 Conclusion

Our analysis showed that the TB epidemic in Ningxia has remained prominent but has gradually decreased from 48.22 (per 100,000 people) in 2005 to 30.47 (per 100,000 people) in 2023. Geographically, high-incidence areas are still concentrated in the relatively poor areas of the southern mountainous regions. Beijing-type MTB is the dominant strain responsible for most patient infections. Molecular epidemiological investigations conducted through spoligotyping and McSpoligotyping showed that the main genotypes have persisted during the TB epidemic. TB has always been a serious social issue, and controlling its spread has become a critical global issue. The results of this study provide a basis for recommendations for TB prevention and control. Further molecular epidemiological studies are required to provide new insights into the persistently high incidence of TB in Ningxia.

## Data Availability

The original contributions presented in the study are included in the article/supplementary material, further inquiries can be directed to the corresponding authors.

## Footnotes

^1^ http://www.pasteur-guadeloupe.fr:8081/SITVIT2/upload

^2^ http://www.pasteur-guadeloupe.fr:8081/SITVIT2

^3^ https://www.miru-vntrplus.org
